# Psychological Distress among family caregivers of persons with Alzheimer's Disease and Related Dementias in Uganda

**DOI:** 10.21203/rs.3.rs-3918857/v1

**Published:** 2024-02-12

**Authors:** Joy Louise Gumikiriza-Onoria, Janet Nakigudde, Bruno Giordani, Roy William Mayega, Martha Sajatovic, Mark Kaddu Mukasa, Dennis Buwembo, Kamada Lwere, Noeline Nakasujja

**Affiliations:** Makerere College of Health Sciences

**Keywords:** Alzheimer's Disease, Related Dementias (ADRD), Caregiver Stress, Psychological Distress, Quality of Life (QoL), Depression, Uganda, Familial Caregiving

## Abstract

**Background:**

Alzheimer's disease and related dementia (ADRD) present growing global health challenges, especially in aging populations such as Uganda. In Uganda, familial caregiving, predominantly undertaken by female relatives, is the primary form of support for patients with ADRD. Cultural stigma around dementia and limited access to support services amplify caregivers' challenges. This study examined psychological distress, depression, and quality of life (QoL) among family caregivers of patients with ADRD in Wakiso District, Uganda.

**Methods:**

This cross-sectional study involved 90 caregivers from three sub-counties in Wakiso selected through purposive sampling to capture diverse experiences. Data were collected using the Kessler Psychological Distress Scale, Caregiver Dementia Quality of Life Measurement Scale, and Center for Epidemiologic Studies Depression Scale, with an 80% response rate achieved through local collaboration. Statistical analyses focused on psychological distress, QoL, and depression.

**Results:**

The study included 82.2% females and 17.8% males, with a median age of 52 years for females and 35 years, respectively. Females were more likely to be single or widowed, whereas males were more likely to be married. The study revealed a high prevalence of psychological distress and depression among caregivers (64.4%) regardless of sex. The analysis indicated that having children was a significant predictor of better QoL (OR 3.04, 95% CI 1.79-5.66, p=0.034) and lower risk of depression (OR 0.10, 95% CI 0.01-0.86, p=0.036). No other sociodemographic factor was significantly associated with health outcomes across the models.

**Conclusion:**

Our findings revealed a heavy burden of psychological distress and depression among Ugandan caregivers of patients with ADRD, highlighting the need for structured support systems, including mental health services and gender-responsive interventions, in low-resource settings.

## Background

Alzheimer's disease and related dementia (ADRD) are significant and growing global health challenges. The prevalence of ADRD is increasing with the aging population worldwide, placing strain on healthcare systems, families, and caregivers (Nicholas et al., 2019). Given the growing significance of ADRD and the pivotal role that caregivers play, understanding the specific challenges and distress they face in this context is crucial.

In Uganda, As in many other African countries, familial caregiving remains the primary means of support for individuals with ADRD (Mwaka et al., 2019). Familial caregivers play an indispensable role in providing emotional, physical, and logistical support. Traditional family structures in Uganda often place responsibility on caring for family members, primarily female relatives, who may have limited formal education and resources to manage the complex needs of individuals with ADRD (Wandera et al., 2017). However, this essential service often comes at considerable personal cost. Caregivers of individuals with ADRD often experience high levels of stress, emotional exhaustion, and psychological distress (Ashrafizadeh et al., 2012: Brodaty & Donkin, 2009). These experiences can be exacerbated in settings with limited access to formal health and social support services, as is the case in many parts of sub-Saharan Africa, including Uganda (Wandera et al., 2017; Agyeman et al., 2016).

Cultural beliefs and stigma associated with dementia in Africa can further compound caregiver distress (Backe et al., 2020; Olayinka & Mbuyi, 2014). Misunderstandings about the nature and causes of dementia can lead to delays in seeking medical advice, isolation, and discrimination (Low and Purwaningrum 2020). Moreover, in settings where resources are constrained, caregivers may face additional challenges such as limited access to accurate information about the disease, lack of appropriate medical care, and financial strain (Adelman et al., 2014). In this study, we aimed to systematically investigate psychological distress and its associated factors, such as depression and Quality of Life (QoL), in family caregivers of patients with ADRD in Uganda.

## Methods

This study was conducted in the Wakiso District, which is situated in Uganda's Central Region. According to the National Population and Housing Census conducted by the Uganda Bureau of Statistics (UBOS) in 2014, the population of the district was estimated to be 2,007,700, with 3.8% of the population aged 65 years and above (UBOS, 2017). In this region as well as in the rest of Uganda, older adults primarily depend on their social networks for both care and economic support. This trend has been widely documented in the literature and the intricacies of these support systems, including their limitations, documented (Golaz, V., Wandera, S. O., & Rutaremwa, G., 2017). Our study was conducted in three representative sub-counties of Wakiso district: Nansana (urban), Busukuma, and Nakwero (rural).

Participant Selection Purposive sampling was used to select 90 caregivers from the specified sub-counties, ascertaining participants with diverse, first-hand experiences as the primary caregivers of patients with ADRD to ensure comprehensive data collection. This method was consciously chosen because of its ability to select participants with diverse experiences and its targeted approach, with specific criteria for age, sex (male and female), and active caregiver status. To enhance effectiveness, we collaborated with Village Health Teams (VHTs) and local council leaders, who identified households with known ADRD patients [who had been diagnosed by a psychiatrist and clinical psychologist in a previous study evaluating the stress and caregiver burden of individuals looking after patients with Alzheimer's disease in the community]. However, to maintain the integrity of the study, we excluded caregivers who were critically ill, those who were not the primary caregivers of the patients, or those below 18 years of age. Through rapport built with local entities and a deliberate selection strategy, we approached 113 homesteads with identified patients with ADRD and achieved an 80% caregiver response rate, underscoring the method's efficacy and alignment with our research objectives.

### Data Collection Procedures

A team of three experienced research assistants with backgrounds in community psychology, social work, and nursing led the data-collection process. The interviews lasted for 90–120 minutes to complete. All the sessions were conducted in Luganda, the dominant local language, to ensure clear communication. To ensure that the study met ethical standards, clearance was obtained from the Makerere School of Medicine Research and Ethics Committee and Uganda National Council for Science and Technology (HS2909ES). Before data collection, each potential participant was approached individually to explain the purpose, nature, potential benefits, and risks of the study. Participants were informed that their participation was voluntary and that they had the right to withdraw without consequences. Written informed consent was obtained from those willing to participate after ensuring their understanding and comfort. Data were stored securely, ensuring limited access to authorized personnel and maintaining the anonymity and privacy of the participants.

Data Collection Instruments Sociodemographic details, including age, sex, educational background, occupation, comorbidities, and marital status, were collected through a brief questionnaire. The Kessler Psychological Distress Scale (K10) was used to assess psychological distress (Kessler et al., 2003. Naisanga et al., 2022), with an internal consistency of 0.78. A cutoff score of 22 indicated severe psychological distress. The Caregiver Dementia Quality of Life Measurement (C-DMEQOL) evaluated caregiver quality of life with an internal consistency of 0.87 and consisting of five key domains. A score of 95 or higher indicates a good quality of life. The Center for Epidemiologic Studies Depression Scale (CES-D) (Miler., Kintu., & Kiene., 2020)was used to assess self-reported depressive symptoms, with an internal consistency of 0.79. Scores of 16 or higher indicate the presence of depression.

### Data Management and Statistical Analysis

A formal analysis of sociodemographic attributes was conducted using frequency distributions, and gender-based differences were examined using chi-square tests. The Mann-Whitney U test was used to investigate age-related disparities by sex. Our study aimed to assess the degree of psychological distress among caregivers of individuals with ADRD while the primary independent variables encompassed potential distress triggers that may involve caregiver demographics. The reliability of the C-DMEQOL, K10-scale, and CES-D was confirmed using Cronbach's alpha. The outcome variables of interest, including the prevalence of psychological distress, quality of life, and depression indicators, were determined using proportions. Multivariate logistic regression analyses were performed to explore the relationships between various factors and outcomes, and both crude and adjusted odds ratios (OR), along with 95% confidence intervals (CI), were computed to identify the strength and orientation of these associations. Statistical significance was set at p < 0.05, and all analytical procedures were performed using the Stata 17.

## Results

Overall Sample Description: This study included 90 participants. Socio-demographic and multivariate logistic regression analyses of participants’ psychological distress, QoL, and depression outcomes were conducted. Table 1 summarizes the sociodemographic characteristics of the participants by sex. The sample consisted of 82.2% females and 17.8% males, with a median age of 52 years for females and 35 years for males (p = 0.052). Females were more likely to be single (34.2%) or widowed (13.7%), whereas males were more likely to be married (56.3%). Females also had lower educational levels, with 52.7% having primary or lower education, compared to 25.0% of males. Most participants had children (87.8% female, 75.0% male) and comorbidities, with HIV being more prevalent in males (62.5%) and UTI in females (59.5%, p = 0.040).

### Scale Scores and Prevalence:

Table 2 displays the average QoL, psychological distress, and depression scores according to sex. No significant disparities in these scores were detected between males and females were detected. The prevalence of psychological distress was 64.4%, and that of QoL revealed a poor QoL (52.2%). Furthermore, 64.4% of the individuals exhibited symptoms of depression.

### Multivariate Linear Regression Models:

Tables 3-5 present the results of the multivariate linear regression analysis of QoL, Psychological Distress, and Depression. The analysis revealed that having children was a significant predictor of better QOL in the adjusted model (OR 3.04, 95% CI 1.79–5.66, p = 0.034). Psychological distress was significantly associated with poorer QOL in the adjusted model for distress outcomes (OR 3.25, 95% CI 1.01–10.82, p = 0.044), and having children predicted lower risk of depression (OR 0.10, 95% CI 0.01–0.86, p = 0.036) in the adjusted depression model. No other socio-demographic factors showed significant associations with health outcomes across the models.

## Discussion

This cross-sectional study aimed to investigated psychological distress and associated factors, such as depression and Quality of Life (QoL), among family caregivers of patients with ADRD in Uganda. We conducted a study on 90 caregivers of patients with ADRD in Wakiso, Uganda, to provide invaluable information regarding their demographic profiles and psychological experiences. The majority of participants were female (82.2%), with a median age of 52 years for females and 35 years for males. The study also found that single female caregivers accounted for 48.0% of the group, whereas married males accounted for 56.3%. Male educational attainment was skewed towards secondary levels, whereas females had a higher prevalence of primary or lower education. The study further highlighted the socioeconomic constraints and health challenges faced by this caregiving group, including comorbidities such as HIV and UTIs, and sources of income. The study further discovered that psychological distress affected 64.4% of caregivers, and quality of life scores were nearly even, emphasizing that significant toll caregiving takes on individuals and the need for attention to both the economic and psychosocial dimensions of caregiver support.

Prevalence of Psychological Distress and Depression: The findings of our study, which indicate a high prevalence of psychological distress (64.4%) and depression (64.4%) among caregivers, are consistent with the existing literature on the impact of caregiving on mental health (Ashrafizadeh et al., 2012; Brodaty & Donkin, 2009). The results of our research highlight a crucial and often-overlooked aspect of the ADRD caregiving experience, particularly in low- and middle-income settings such as Uganda. The significant proportion of caregivers who reported symptoms of psychological distress and depression (64.4%) highlighted a substantial mental health challenge within the Wakiso community. This prevalence is consistent with previous research in different settings, underscoring the significant psychological toll that caregiving can have on individuals (Ashrafizadeh et al., 2012; Brodaty & Donkin, 2009).

Furthermore, the high comorbidity of psychological distress and depression among caregivers highlights the multifaceted nature of their burden. Caregivers not only face the emotional strain of witnessing the progressive decline of their loved ones but are also subject to the tangible hardships of providing care, often in environments with limited resources and support (George et al., 2020; Lindeza et al., 2020; Shah., Wadoo., & Latoo,.2010). The confluence of these challenges is potentially detrimental, increasing caregivers’ susceptibility to mental health issues. Ashrafizadeh et al. (2012) highlight the diverse sources of stress experienced by caregivers, including the physical demands of caregiving, the emotional challenge of dealing with behavioral symptoms of ADRD, and the economic strain of shouldering medical costs. Brodaty and Donkin (2009) further noted that in the absence of adequate support structures, these sources of stress are likely to coalesce, intensifying the risk of psychological distress and depression among caregivers.

### Gender Differences:

A significant proportion of the participants were females (82.2%). This highlights the traditional caregiving role that women often shoulder, particularly in LMICs (Cohen et al 2019). Despite the gender skew, the study did not find significant disparities in Quality of Life, Psychological Distress, or depression between the genders. This possibly indicates that the act of caregiving, not gender, is the primary driver of the psychological challenges faced. That said, socioeconomic conditions affecting genders differently, such as females predominantly relying on relatives for income and males relying on business activities, might intersect with caregiving roles in subtle, yet crucial ways.

### Quality of Life & Age:

Contrary to expectations, this study did not find a statistically significant association between age and Quality of Life (QoL). The results indicated that age, whether lower or higher, did not significantly impact QoL among the caregivers. This suggests that the challenges impacting caregivers' QoL may be more universally experienced across different age groups rather than being particularly pronounced in younger caregivers. While previous research has highlighted the dual stress faced by younger caregivers in balancing caregiving responsibilities with their personal and professional lives (CDC 2023; Cheng et al 2022; Leocadie et al 2020; Lindeza et al 2020), our findings suggest that this stress does not significantly differentiate QoL across age groups. This may indicate the need to consider factors other than age when addressing QoL among caregivers.Quality of Life & Age: Interestingly, this study found a lower age to be associated with a decrease in QoL. This implies that younger caregivers, possibly in their prime working years, face the dual stress of caregiving and managing their personal and professional lives. This aligns with the hypothesis that caregivers’ QoL is inversely correlated with distress. Furthermore, this finding aligns with existing literature. For instance, research has shown that younger caregivers often experience the dual stress of managing their caregiving responsibilities along with their personal and professional lives (CDC 2023; Cheng et al 2022; Leocadie et al 2020; Lindeza et al 2020). This could be particularly challenging during their prime working years as career development and personal growth are crucial.

### Children and Psychological Distress:

The finding that having fewer children is linked to decreased psychological distress is intriguing and noteworthy in the caregiving context. Initially, one might assume that having children equates to increased support and reduced stress. However, the dual responsibility of caregiving for a loved one and parenting can be overwhelming, resulting in increased stress. Folkman and Lazarus (1988) identified role conflicts as a source of stress, as individuals struggling to balance competing demands. Pearlin et al. (1990) demonstrated that caregivers who take on multiple roles are susceptible to role strain, leading to feelings of inadequacy and heightened psychological distress. Given the complexity of caregiving and the need to attend to the needs of multiple children, caregivers with more children may be particularly vulnerable to this strain.

Implications for Policy and Practice: The results have profound implications for the development of healthcare policies and support services in Uganda and similar LMIC settings. There is a pressing need for structured caregiver support systems that incorporate mental health screenings, counselling services, and community-based respite care programs. Given the gendered nature of caregiving, gender-responsive interventions that recognize the unique challenges faced by male and female caregivers could be beneficial.

### Strengths and Limitations

This study offers a fresh perspective on a previously under- researched demographic. Rigorous methodology, including purposive sampling and the use of standardized scales with proven internal consistency, underpins the strengths of the study. However, its cross-sectional nature prevents causal inferences. The focus on one district, although detailed, might not be representative of the entire Ugandan context.

## Conclusion

Amidst the challenges presented by Alzheimer's Disease and Related Dementias, caregivers, often overlooked, bear a substantial emotional and psychological burden. The intersection of gender, age, socioeconomic status, and other factors with caregiving in the Ugandan context provides a nuanced understanding of caregivers' challenges. These results underscore the pressing need for community, healthcare, and policy interventions. Addressing caregivers' psychological well-being is not just an act of empathy; it is also a critical component in the holistic care of patients with ADRD.

## Figures and Tables

**Table 1:** Socio-Demographics Characteristics

Factor	Gender		p-value
	Male (n = 16; 17.8%)	Female (n = 74; 82.2%)	
Age (years)	35 (23,43)	52 (37,61)	0.052
Marital status			
Widow	0 (0.0%)	10 (13.7%)	0.231
Married	9 (56.3%)	25 (34.2%)	
Divorced	1 (6.3%)	3 (4.1%)	
Single	6 (37.5%)	35 (48.0%)	
Education level			
Primary & below	4 (25.0%)	39 (52.7%)	0.115
Secondary	10 (62.5%)	31 (41.9%)	
Tertiary	2 (12.5%)	4 (5.4%)	
Children			
No	4 (25.0%)	9 (12.2%)	0.185
Yes	12 (75.0%)	65 (87.8%)	
Comorbidity			
HIV	10 (62.5%)	29 (39.2%)	0.088
UTI	5 (31.3%)	44 (59.5%)	0.040
Source of income			
None	8 (50.0%)	35 (47.3%)	0.119
Farming	1 (6.3%)	15 (20.3%)	
Relatives	0 (0.0%)	10 (13.5%)	
Business	6 (37.5%)	11 (14.9%)	
Professional job	1 (6.3%)	3 (4.1%	
Monthly income			
100,000 & below	10 (62.5%)	61 (82.4%)	0.076
Over 100,000	6 (37.5%)	13 (17.6%)	

**Table 2:** Scale scores and prevalences

Factor	FREQUENCY (PERCENTAGE)	Gender	
		Male (n = 16)	Female (n = 74)
**QOL**			
Poor	47 (52.2%)	8 (50%)	39 (52.7%)
Good	43 (47.8%)	8 (50%)	35 (47.3%)
**Psychological distress**			
No/mild distress	32 (35.6%)	4 (25.0%)	28 (37.8%)
Moderate/severe distress	58 (64.4%)	12 (75.0%)	46 (62.2%)
**Depression**			
No Depression	32(35.6%)	3 (18.75%)	12 (16.2%)
Symptomatic Depression	58 (64.4%)	13 (81.25%)	62 (83.8%)

**Table 3:** Results for fitting a multivariate logistic regression models. (Outcome : QOL)

Factor	level	Crude estimates		Adjusted model	
		Odds ratio (95% CI)	p- value	Odds ratio (95% CI)	p- value
Age	Mean (SD)	0.99 (0.97,1.01)	0.354	0.96 (0.93,1.00)	0.076
Gender	Male	Reference			
	Female	0.90 (0.30,2.65)	0.844	0.55 (0.10,2.90)	0.481
Marital status	Widow	Reference			
	Married	1.27 (0.31,5.20)	0.588	1.14 (0.20,6.51)	0.523
	Divorced	-		-	
	single	0.78 (0.20,3.13)		0.57 (0.09,3.41)	
Education level	Primary & below	Reference			
	Secondary	0.82 (0.35,1.94)	0.699	1.05 (0.30,3.67)	0.544
	Tertiary	0.48 (0.08,2.89)		0.25 (0.02,3.29)	
Children	No	Reference			
	Yes	2.56 (1.47,5.19)	0.045	3.04 (1.79,5.66)	0.034
Comorbidity	HIV	1.54 (0.66,3.56)	0.315	1.94 (0.61,4.56)	0.315
	UTI	0.78 (0.34,1.78)	0.550	0.58 (0.34,2.78)	0.550
Source of income	None	Reference	0.552		
	Farming	1.35 (0.42,4.27)		1.51 (0.37,6.23)	0.536
	Relatives	0.45 (0.10,1.97)		0.44 (0.08,2.56)	
	Business	0.73 (0.24,2.28)		0.63 (0.11,3.55)	
	Professional job	3.14 (0.30,32.65)		5.85 (0.18,187.42)	
Monthly income	100,000 & below	Reference			
	Over 100,000	0.42 (0.14,1.24)	0.117	0.14 (0.02,1.01)	0.051
Psychological distress	No/mild distress	Reference			
	Moderate/severe distress	1.91 (0.79,4.62)	0.149	3.14 (0.96,10.29)	0.058
Depression	No Depression	Reference			
	Depression	2.74 (0.68,11.07)	0.158	2.39 (0.48,11.96)	0.288

**Table 4:** Results for fitting a multivariate logistic regression models. (Outcome: Psychological distress

Factor	level	Crude estimates		Adjusted model	
		Odds ratio (95% CI)	p-value	Odds ratio (95% CI)	p-value
Age	Mean (SD)	1.00 (0.98,1.03)	0.731	1.01 (0.97,1.05)	0.625
Gender	Male	Reference			
	Female	0.55 (0.16,1.86)	0.335	0.67 (0.144,3.09)	0.606
Marital status	Widow	Reference			
	Married	0.6 (0.11,3.34)	0.375	0.50 (0.08,3.25)	0.469
	Divorced	0.25 (0.02,3.04)		1.12 (0.05,22.93)	
	single	0.32 (0.06,1.69)		0.30 (0.05,1.97)	
Education level	Primary & below	Reference			
	Secondary	0.75 (0.31,1.85)	0.820	0.53 (0.16,1.81)	0.510
	Tertiary	0.97 (0.16,5.92)		1.29 (0.11,15.08)	
Children	No	Reference			
	Yes	0.28 (0.06,1.38)	0.118	0.09 (0.01,0.67)	0.019
Comorbidity	HIV	0.66 (0.28,1.57)	0.344	-	-
	UTI	1.32 (0.56,3.14)	0.530	-	-
Source of income	None	Reference			
	Farming	0.38 (0.12,1.22)	0.367	0.37 (0.91,1.53)	0.514
	Relatives	0.72 (0.18,2.99)		0.92 (0.15,5.64)	
	Business	1.57 (0.43,5.70)		1.97 (0.30,13.07)	
	Professional job	1.45 (0.14,15.21)		2.50 (0.14,45.60)	
Monthly income	100,000 & below	Reference			
	Over 100,000	1.72 (0.56,5.31)	0.347	1.43 (0.24,8.47)	0.695
QOL	Good	Reference			
	Poor	1.91 (0.79,4.62)	0.149	3.25 (1.01,10.82)	0.044
Depression	No depression	Reference			
	depression	1.60 (0.45,5.74)	0.467	1.05 (0.22,4.94)	0.947

**Table 5:** Results for fitting a multivariate logistic regression model. (outcome: Depression)

Factor	Level	Crude estimates		Adjusted model	
		Odds ratio (95% CI)	p- value	Odds ratio (95% CI)	p- value
Age	Per unit increase	0.99 (0.96,1.01)	0.317	0.98 (0.94,1.02)	0.335
**Gender**	Male	**Reference**			
	Female	0.79 (0.25,2.52)	0.692	1.26(0.28,5.75)	0.762
**Marital status**	Widow	**Reference**			
	Married	2.17(0.49,9.64)	0.750	2.56(0.45,14.42)	0.185
	Divorced	0.67(0.06,6.87)		**2.53(0.14,46.12)**	
	single	0.85(0.21,3.48)		0.60(0.11,3.38)	
**Education level**	Primary & below	**Reference**			
	Secondary	1.03(0.42,2.54)	0.811	0.56(0.16,1.95)	0.179
	Tertiary	0.54(0.09,3.00)		0.05(0.01,1.22)	
**Children**	No	**Reference**			
	Yes	0.28(0.06,1.37)	0.118	0.10(0.01,0.86)	0.036
**Comorbidity**	HIV	0.97(0.41,2.33)	0.953	-	-
	UTI	0.89(0.37,2.13)	0.798	-	-
**Source of income**	None	**Reference**			
	Farming	1.74(0.51,5.88)	0.481	4.48(0.91,22.0)	0.170
	Relatives	1.19(0.29,4.82)		1.10(0.20,6.21)
	Business	2.57(0.72,9.18)		4.01(0.63,25.45)
	Professional job	-		-
**Monthly income**	100,000 & below	**Reference**			
	Over 100,000	1.72(0.56,5.31)	0.347	0.63(0.10,3.96)	0.621
**Quality of life**	Good	**Reference**			
	Poor	1.29(0.54,3.06)	0.170	3.45 (0.64,8.70)	0.015
**Psychological distress**	No/mild distress	**Reference**			
	Moderate /severe disorder	2.13(1.13,5.21)	0.038	2.06 (1.21,5.28)	0.021

## Abbreviations

ADRD: Alzheimer's Disease and Related Dementias
QoL: Quality of Life
K10: Kessler Psychological Distress Scale
C-DMEQOL: Caregiver Dementia Quality of Life Measurement
CES-D: Center for Epidemiologic Studies Depression Scale
OR: Odds Ratio
CI: Confidence Interval
UBOS: Uganda Bureau of Statistics
VHTs: Village Health Teams
